# A small-molecule inhibitor targeting the AURKC–IκBα interaction decreases transformed growth of MDA-MB-231 breast cancer cells

**DOI:** 10.18632/oncotarget.18883

**Published:** 2017-06-29

**Authors:** Eun Hee Han, Jin-Young Min, Shin-Ae Yoo, Sung-Joon Park, Yun-Jeong Choe, Hee Sub Yun, Zee-Won Lee, Sun Woo Jin, Hyung Gyun Kim, Hye Gwang Jeong, Hyun Kyoung Kim, Nam Doo Kim, Young-Ho Chung

**Affiliations:** ^1^ Drug & Disease Target Research Team, Division of Bioconvergence Analysis, Korea Basic Science Institute (KBSI), Cheongju 28119, South Korea; ^2^ Immunotherapy Convergence Research Center, Korean Research Institute of Bioscience and Biotechnology (KRIBB), Daejeon 34141, South Korea; ^3^ Graduate School of Analytical Science and Technology (GRAST), Chungnam National University, Daejeon 34134, South Korea; ^4^ Department of Toxicology, College of Pharmacy, Chungnam National University (CNU), Daejeon 34133, South Korea; ^5^ New Drug Development Center, Daugu Gyeoungbuk Medical Innovation Foundation (DGMIF), Daegu 41061, South Korea; ^6^ Department of Bioanalytical Science, Korea University of Science and Technology (UST), Daejeon 34113, South Korea

**Keywords:** AURKC, protein–protein interaction, IκBα, small-molecule inhibitor, breast cancer

## Abstract

The Aurora kinases, Aurora A (AURKA), Aurora B (AURKB), and Aurora C (AURKC), are serine/threonine kinases required for the control of mitosis (AURKA and AURKB) or meiosis (AURKC). Several Aurora kinase inhibitors are being investigated as novel anticancer therapeutics. Recent studies demonstrated that AURKC activation contributes to breast cancer cell transformation. Therefore, AURKC is both a promising marker and therapeutic target for breast cancer; however, its signaling network has not been fully characterized. Using translocation-based cellular assays, we identified IκBα as a binding partner of AURKC, and found that AURKC phosphorylates IκBα at Ser32, thereby activating it. *In silico* modeling and computational analyses revealed a small-molecule inhibitor (AKCI) that blocked the AURKC–IκBα interaction and exerted antitumor activity in MDA-MB-231 breast cancer cells. Specifically, AKCI induced G2/M cell-cycle arrest through modulation of the p53/p21/CDC2/cyclin B1 pathways. In addition, the drug significantly inhibited MDA-MB-231 cell migration and invasion, as well as decreasing colony formation and tumor growth. Via its interaction with IκBα, AURKC indirectly induced NF-κB activation; accordingly, AKCI decreased PMA-induced activation of NF-κB. Thus, the small-molecule inhibitor AKCI represents a first step towards developing targeted inhibitors of AURKC protein binding, which may lead to further advances in the treatment of breast cancer.

## INTRODUCTION

Breast cancer is the most lethal of the female-specific malignancies, as well as the most frequent cancer, with the second-highest mortality rate of all cancers in women worldwide [1]. The World Health Organization estimates that more than 1.2 million people are diagnosed with breast cancer each year [2]. Standard breast cancer therapy generally involves a combination of surgery, multi-therapeutic agents, and ionizing radiation [3].

Most current anticancer agents induce cell-cycle arrest and/or cell death via apoptotic or non-apoptotic mechanisms, including necrosis, senescence, autophagy, and mitotic catastrophe [4]. The Aurora kinases, a family of oncogenic serine/threonine kinases involved in the mitotic (M) phase of the cell cycle, participate in establishment of the mitotic spindle, bipolar spindle formation, alignment of centrosomes on the mitotic spindle, centrosome separation, cytokinesis, and monitoring of the mitotic checkpoint [3, 4–7]. These proteins are critical for accurate and organized division and segregation of chromosome to the daughter cells [7]. Consistent with their roles in promoting mitosis, they are often overexpressed in tumor cells, particularly those with high growth fractions [8]. In humans, three Aurora kinases (A, B, and C) are expressed in neoplastic and non-neoplastic tissues. Aurora A and B kinases (AURKA and AURKB) are expressed globally in all tissues, whereas Aurora C kinase (AURKC) is primarily expressed in testes, where it participates in meiosis [8]. Currently, several selective and nonselective Aurora kinase inhibitors are being tested in preclinical and clinical trials as antitumor agents [9]. Recent studies linked AURKC activity to tumorigenesis in somatic tissue, indicating that it may be a relevant cancer target [10, 11]. AURKC induces abnormal cell division in cell lines, as well as tumor formation in nude mice [12]. Breast tumors often significantly overexpress AURKC in comparison with normal breast tissues [13]. Although AURKC is a potential anticancer drug target, no AURKC-specific inhibitors are currently in development, limiting our ability to elucidate AURKC-specific anticancer effects.

Protein–protein interactions (PPIs) are of pivotal importance in regulation of biological systems and their interfaces represent a highly promising, although challenging, class of potential targets for drug development [14–16]. In cancer, PPIs are signaling nodes and hubs that transmit pathophysiological cues, so efforts are constantly being made to target drugs, particularly PPIs [14]. Several small-molecule inhibitors that target allosteric PPI hotspots have recently been reported [16]. For example, PS210, identified by virtual screening for molecules targeting PDK1, can inhibit the PDK1/PIFtide interaction. ABT199, which targets the BCL2/Bcl-xL interaction, is currently in phase 1 trials for chronic lymphocytic leukemia (CLL) [17].

In this study, we identified IκBα as a PPI partner protein of AURKC, as well as a small-molecule inhibitor that targets the AURKC–IκBα interaction and interferes with the growth and tumorigenic activity of breast cancer cells.

## RESULTS

### Identification of AURKC/IκBα molecular interactions

**Figure 1:**
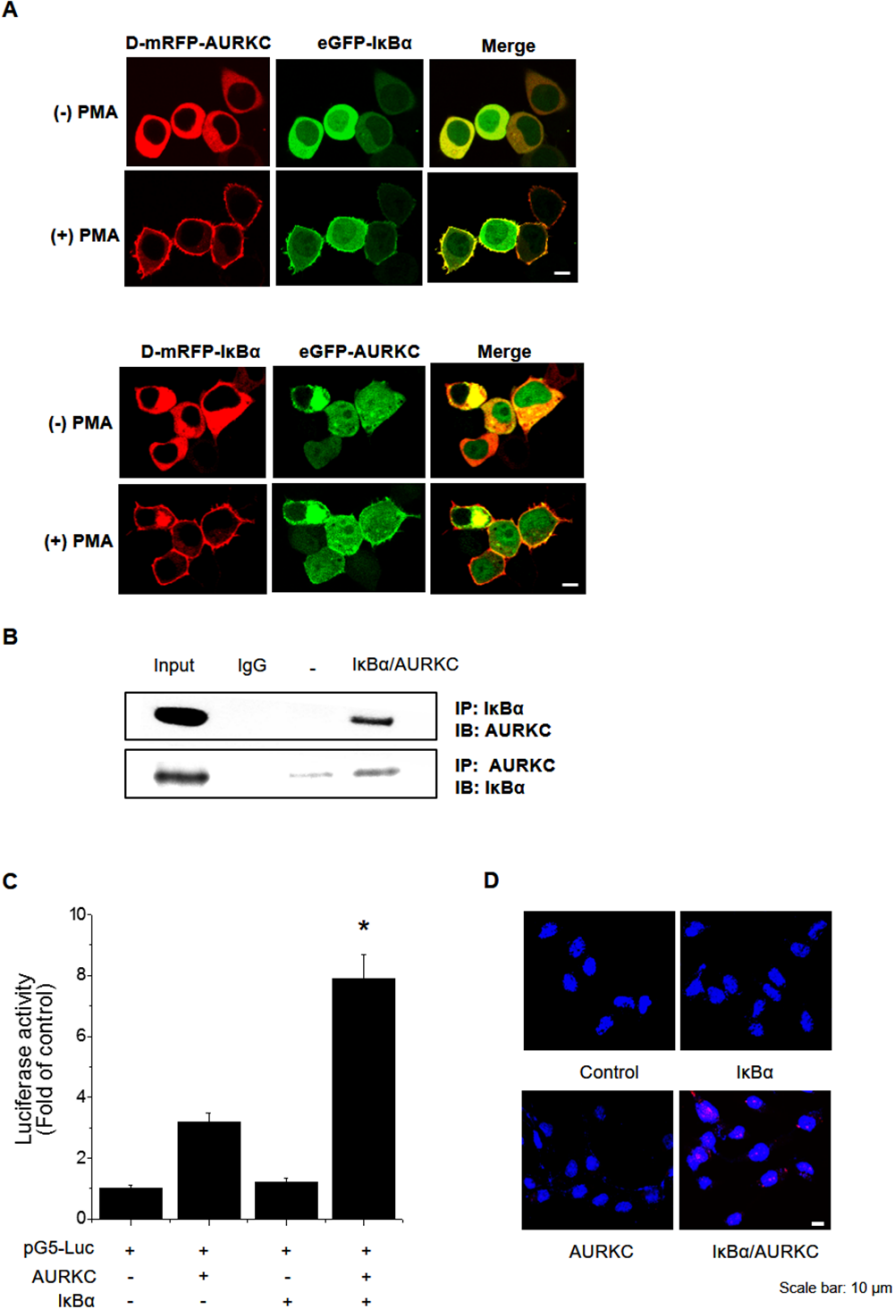
Identification and confirmation of the AURKC–IκBα interaction **(A)** IκBα is a novel AURKC-interacting protein. HEK293T cells were cotransfected with PKCδ–mRFP–AURKC or PKCδ–mRFP–IκBα (bait) and eGFP–IκBα or eGFP–AURKC (prey). Before PMA treatment, the mRFP-tagged bait and GFP-tagged prey proteins were localized in the cytoplasm. However, after PMA (300 nM) was added, bait and prey proteins were relocalized to the plasma membrane due to the translocation properties of PKCδ. Scale bar, 10 μm. **(B)** Immunoblotting to confirm the interaction. Whole-cell lysates of HEK293T cells transfected with AURKC and IκBα were subjected to immunoprecipitation with either IgG (negative control) or anti-AURKC antibody, followed by Western blot of the immunoprecipitates with anti-IκBα antibody. **(C)** Interaction between proteins expressed from pM-BD-pAURKC and pVP-IκBα was assessed using the mammalian two-hybrid (M2H) assay. Luciferase activity indicates the change in relative luminescence units, normalized against the negative control. * P < 0.01, significantly different from control as determined by analysis of variance (Newman–Keuls test). **(D)** PLA for detection of binding of AURKC and IκBα in HEK293T cells, performed using the Duo-Link kit (magnification, 40×; scale bar, 10 μm). Nuclei are stained with DAPI (blue); Duo-Link signals are shown in red. Each red dot represents a single AURKC–IκBα molecular interaction event.

To identify the binding partner of AURKC, we performed a cellular protein translocation–based screen using a GFP-tagging vector library in HEK293T cells. The library consists of GFP fusions of 597 kinases and kinase-related genes, and was designed for analysis of PPI in cells by laser scanning fluorescence microscopy [18, 19]. We focused the screen on kinases because they represent a set of readily druggable targets related to AURKC that are potentially amenable to clinical translation. Before PMA treatment, AURKC and IκBα were localized in the cytoplasm. Following treatment with 300 nM PMA, both proteins relocalized to the plasma membrane due to the translocation properties of PKCδ. We found that IκBα interacted with AURKC, as demonstrated by an increase in the ratio of cellular translocation in GFP-IκBα–transfected versus control vector–transfected wells (Figure 1A, upper images). To validate AURKC–IκBα binding, we performed transfection in the opposite manner, i.e., with GFP-AURKC and PKCδ–mRFP–IκBα (Figure 1A, lower images). In addition, we confirmed the AURKC–IκBα interaction using the same technique in CHO-K1 cells (Supplementary Figure 1). Furthermore, we evaluated the specificity of the AURKC–IκBα interaction by individually replacing the plasmids encoding each partner with empty vector (Supplementary Figure 2A, 2B, 2C).

To confirm the physical interaction between AURKC and IκBα, we performed co-immunoprecipitation (co-IP) experiments using whole-cell extracts from HEK293T cells. Lysates from cells overexpressing full-length AURKC and IκBα were immunoprecipitated with IκBα or AURKC antibody or normal IgG, and the immunoprecipitates were subjected to 10% SDS-PAGE and Western blot analysis with anti-AURKC and anti-IκBα antibodies. As shown in Figure 1B, IκBα and AURKC reciprocally co-precipitated in HEK293T cells when using a specific antibody against either protein, but not normal IgG. To further confirm the interaction, we performed a mammalian two-hybrid assay using the pGC-luc, Bind-AURKC, and Act-IκBα plasmids. Luciferase activity, representing binding of AURKC and IκBα, was about 2.7-fold higher than that of the Bind-AURKC vector (Figure 1C). This result indicated that AURKC interacts with IκBα in mammalian cells. Furthermore, to confirm the binding of AURKC and IκBα *in vivo*, we performed a proximity ligation assay (PLA) using the Duo-Link kit. Interactions between AURKC and IκBα appeared as red dots in cellular images. Red dots were not detected in untreated controls or in cells treated with one antibody alone. By contrast, in samples exposed to both kinds of antibodies, red dots were observed, confirming the interaction between the two proteins (Figure 1D). Comparisons of the counts of red dots in images confirmed that AURKC bound IκBα in HEK293T cells. Taken together, these binding analysis results provide evidence supporting an interaction between AURKC and IκBα *in vitro* and *in vivo*.

### IκBα is involved in AURKC-mediated transformation in invasive breast cancer cells

**Figure 2:**
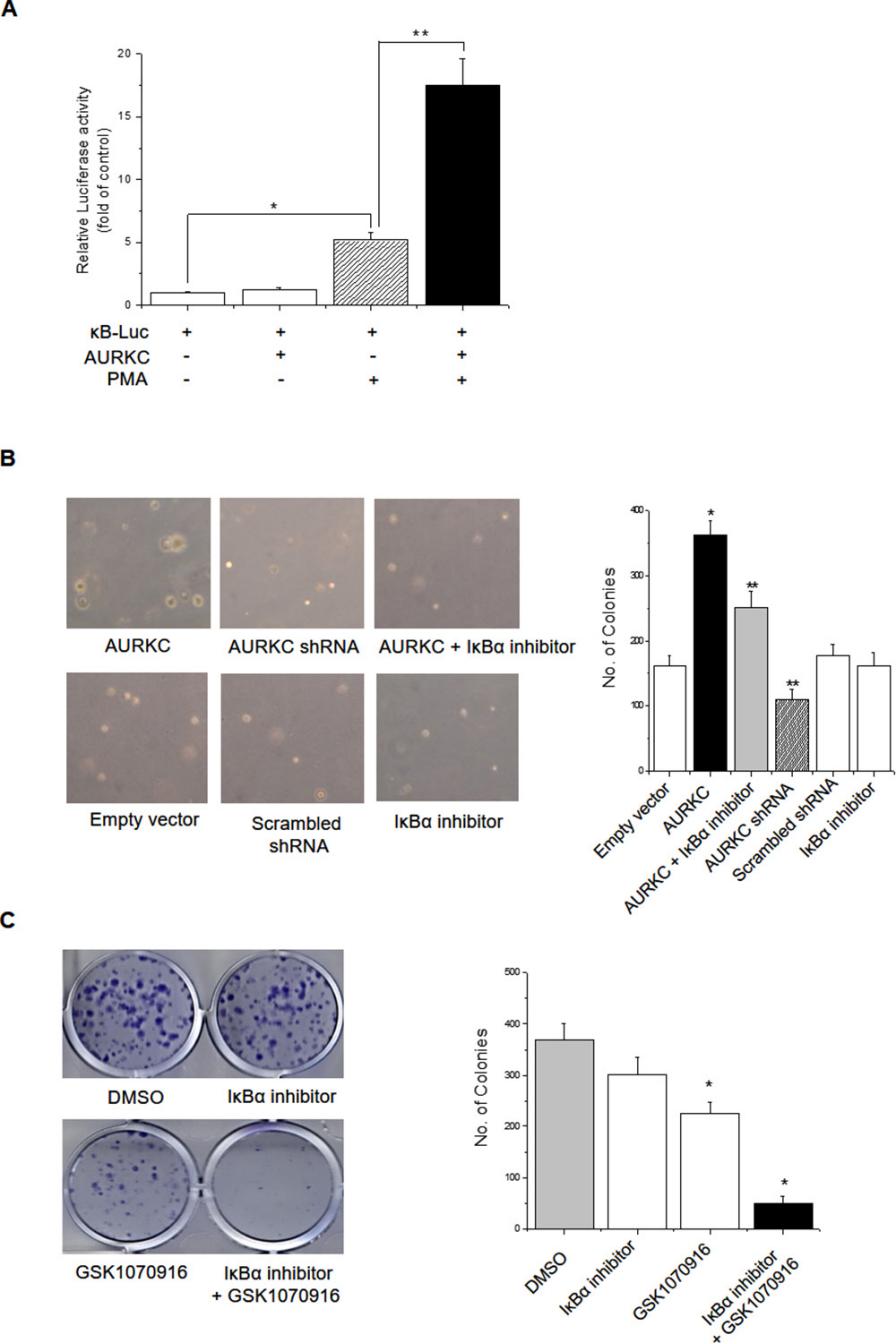
Effects of the AURKC–IκBα interaction on cellular transformation in breast cancer cells **(A)** HEK293T cells were transiently cotransfected with 0.5 μg of pGL3-NF-κB-luc, 0.5 μg of pcDNA3.1-AURKC, and 0.2 μg of pCMV-β-gal. After 4 h, the cells were treated with PMA (100 nM) for 24 h, and luciferase activity was normalized against β-galactosidase activity. * P < 0.01 and ** P < 0.01, significantly different from control and PMA treatment, respectively. **(B)** Empty vector and AURKC stable MDA-MB-231 cell lines (1 × 10^3^ cells/ml) were mixed with 0.3% soft agar and grown on a 0.6% agarose base layer. Anchorage-independent colony formation was decreased by AURKC shRNA (stable cell lines #2 and #3) and IκBα inhibitor treatment. The number of colonies ≥50 μm in diameter was counted 10 days after plating. *Left panel*, images of representative wells; *right panel*, graph. * P < 0.01, significantly different from control as determined by analysis of variance (Newman–Keuls test). **(C)** The tumorigenic effect of AURKC and IκBα on colony formation of MDA-MB-231 cells. Cells were treated with IκBα inhibitor (100 nM) or GSK1070916 (1 nM) for 8 days. Representative images of colony-forming assay and analysis of colony formation rates are shown. Data are means ± SD of three independent experiments. * P < 0.01 vs. control group.

Nuclear factor-κB (NF-κB) is a family of highly regulated dimeric transcription factors that play pivotal roles in cancer cell transformation. IκBα was originally thought to retain NF-κB dimers in the cytoplasm by masking their nuclear localization sequences (NLSs) [20]. Aberrant NF-κB activation in tumor cells results from genetic changes or activation of NF-κB pathways by indirect (i.e., IκBα-mediated) mechanisms. We next examined the functional significance of the interaction between AURKC and IκBα. AURKC is overexpressed in invasive breast cancer cells and exerts oncogenic activity [10, 11], but the precise underlying mechanisms remain unknown. Because IκBα is a binding partner of AURKC, as demonstrated above, we hypothesized that AURKC-induced IκBα activation modulates NF-κB activity. The results of NF-κB promoter–reporter assays revealed that AURKC overexpression did not induce NF-kB activation; however, PMA-induced NF-κB promoter activity was elevated in AURKC-overexpressing cells (Figure 2A). Thus, IκBα might be involved in AURKC-induced transformation in AURKC-overexpressing breast cancer cells. To test this idea, we established AURKC- and shRNA-AURKC–expressing MDA-MB-231 cells using a lentivirus system, and subjected them to soft agar assays. MDA-MB-231 is an invasive breast cancer cell line, and its growth rate increases rapidly when AURKC is overexpressed (data not shown). Induced anchorage-independent growth was more prominent in AURKC-expressing MDA-MB-231 cells than in empty vector and scrambled shRNA overexpressing cells. In addition, anchorage-independent growth was reduced in both AURKC-overexpressing MDA-MB-231 cells treated with IκBα inhibitor and AURKC-knockdown cells (Supplementary Figure 4 for validation of AURKC knockdown by shRNA) (Figure 2B). To further support our observations, we performed colony-forming assays using IκBα inhibitor and the reference compound GSK1070916, an ATP-competitive inhibitor of AURKC. To confirm the synergistic effect of the two inhibitors, cells were treated at 1/10th of the respective inhibitory concentrations of each compound. When both inhibitors were present, the number of colonies was much smaller than when cells were treated with either compound alone (Figure 2C). These results confirmed our hypothesis that IκBα is related to the AURKC-induced transformation in MDA-MB-231 breast cancer cells.

### AURKC phosphorylates IκBα on S32 and binds its ankyrin repeat domain

**Figure 3:**
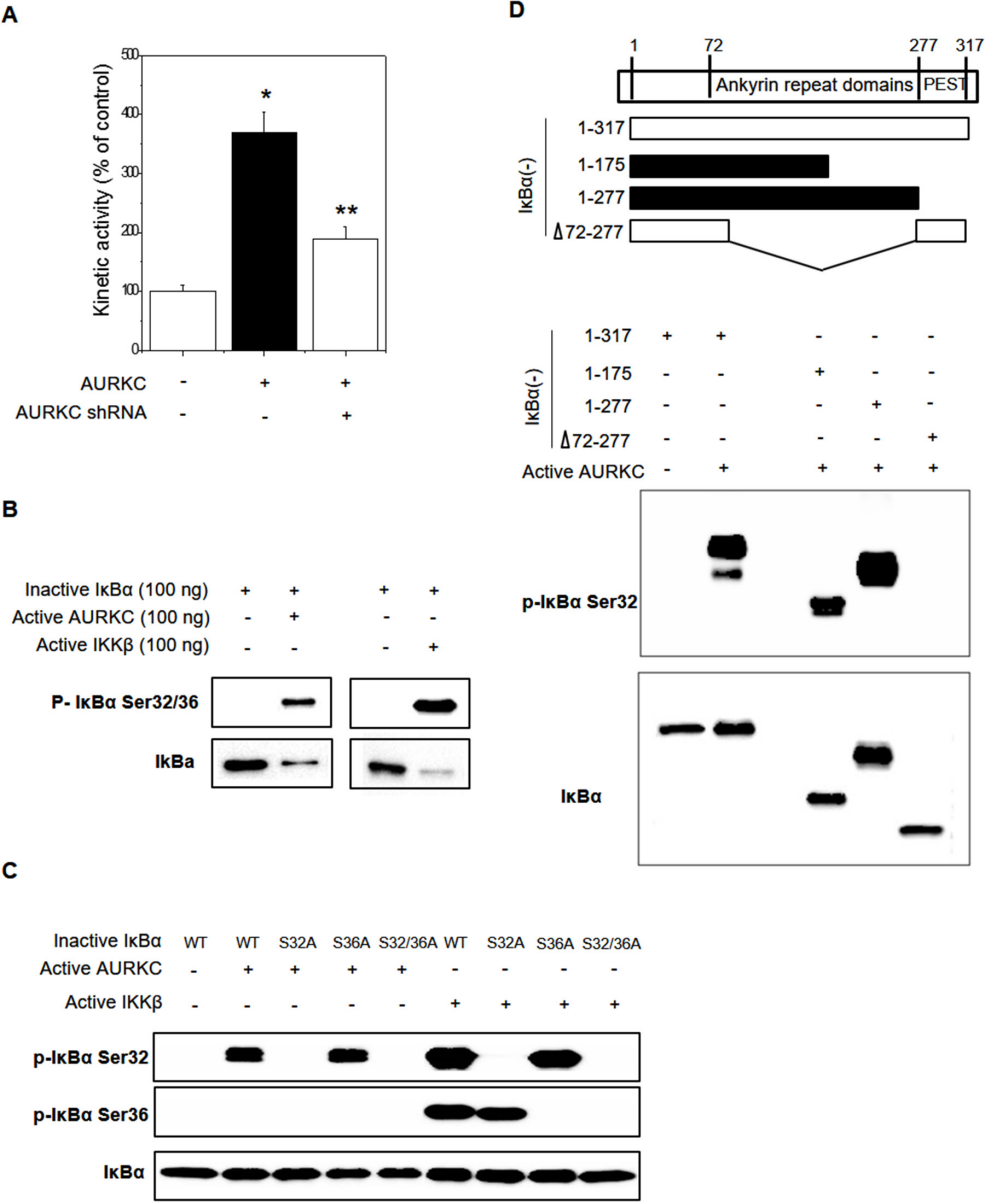
Effects of AURKC on IκBα activation **(A)** Cell-based IκBα activation assay. HEK293T cells were seeded in black 96-well plates and then transfected with AURKC expression vector or shRNA (CCACGATAATAGAGGAGTTGGCAGATGCC) for 24 h. * P < 0.01 and ** P < 0.01, significantly different from control and AURKC as determined by analysis of variance (Newman–Keuls test). **(B)** Purified inactive IκBα protein (WT, S32A, S36A, S32/36A mutant) and active AURKC or IKKβ protein were incubated for 30 min, and then immunoblotted with IκBα S32 and S36 phospho-specific antibodies, as indicated. **(C)** Identification of the interacting domains of AURKC and IκBα. Full-length IκBα and various fragments (top) were purified and incubated with active AURKC protein for 30 min, and then immunoblotted with IκBα S32 phospho-specific antibody. **(D)** Purified inactive IκBα protein (WT, 1–172 aa, 1–277 aa, and 1–72/278–317 aa deletion mutant) and active AURKC protein were incubated for 30 min, and then immunoblotted using an IκBα S32 phospho-specific antibody.

Because AURKC is a serine-threonine kinase, we hypothesized that phosphorylation might modulate the AURKC–IκBα interaction, and in particular that AURKC might activate IκBα. Phosphorylation of IκBα at S32/S36 precedes its dissociation from p65 NF-κB, allowing it to translocate into the nucleus and activate transcription from target promoters. Cell-based phospho-IκBα ELISA revealed that AURKC activated IκBα, whereas AURKC shRNA decreased IκBα activity, in HEK293 cells (Figure 3A). To investigate the precise mechanism, we performed *in vitro* protein kinase assays with activated AURKC kinase and purified IκBα protein using the HaloTag system (Promega). IκBα phosphorylation was increased by active AURKC, and this phosphorylation was slightly lower than IKKβ with known IκBα activator (Figure 3B). As shown in Figure 3C, AURKC induced phosphorylation of the IκBα mutant S36A, but not S32A or the S32/36 dual mutant. Therefore, IκBα phosphorylation in S32 is important for the interaction with AURKC protein. As a positive control, we used IKKβ, which phosphorylates IκBα on serine 32 and 36. These results indicate that AURKC induces site-specific phosphorylation of IκBα.

To identify the interacting domains of IκBα and AURKC, we designed various deletion constructs of IκBα. Plasmids encoding the corresponding fragments of IκBα were constructed, and each IκBα fragment was purified using the HaloTag system. *In vitro* protein kinase assays revealed that AURKC phosphorylated full-length IκBα (1–317 aa) as well as the 1–175 aa and 1–277 aa truncations. However, an internal deletion of IκBα (1–72/278–317 aa) was not phosphorylated by AURKC (Figure 3D). Therefore, the region containing residues 72–175 of IκBα (ankyrin repeat domains 1–3) is important for phosphorylation by AURKC.

### His164 and Arg165 of AURKC interact with the IκBα ankyrin repeat domain

**Figure 4:**
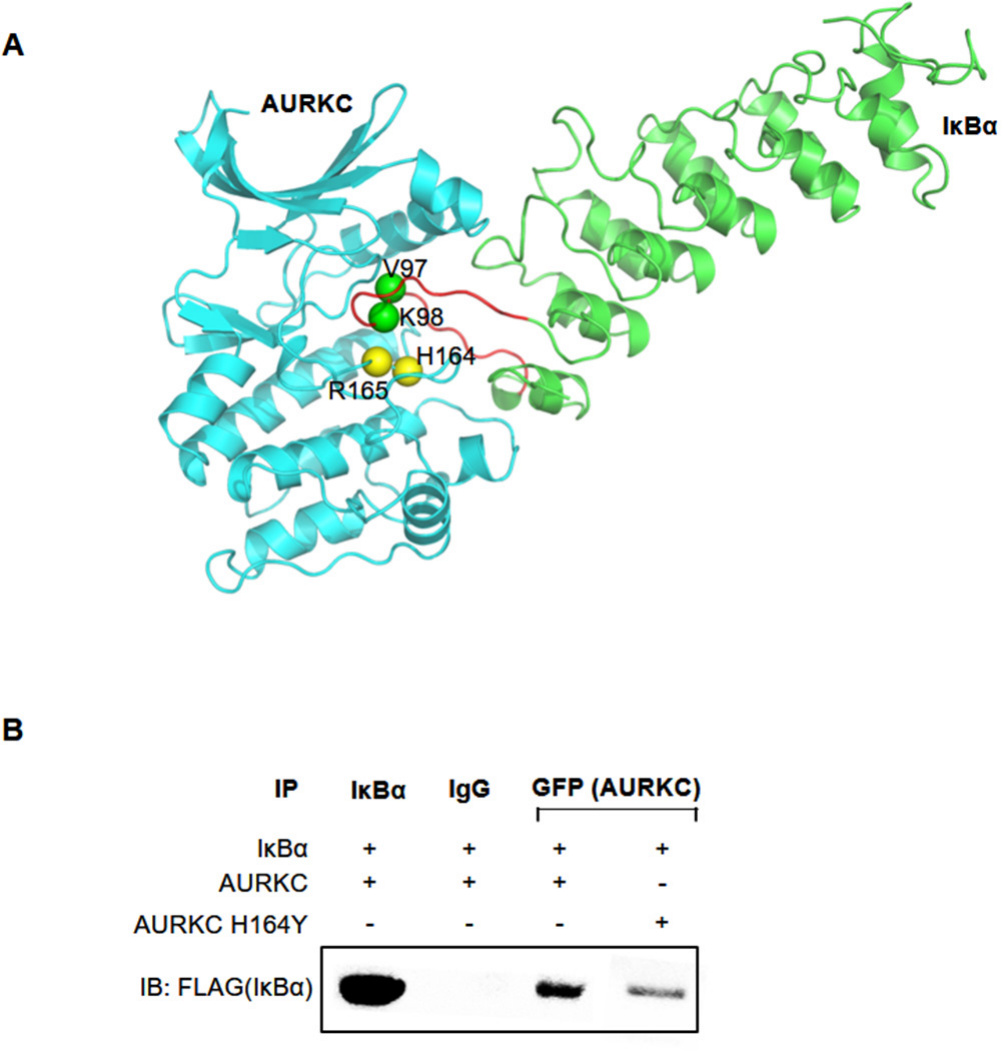
His164 of AURKC interact with IκBα **(A)** Protein–protein interaction model of AURKC and IκBα. The loop from Met 91 to F109 of IκBα is highlighted in red. Green spheres denote V97 and K98 of IκBα, and yellow spheres indicate H164 of AURKC. **(B)** Results of a co-IP assay between IκBα and wild-type AURKC or the AURKC H164Y mutant. Representative blots are shown, and the results of three replications are quantified in the graph.

A model of the AURKC–IκBα complex obtained from protein–protein docking simulations suggested that AURKC bound to the region containing residues 73–175 of IκBα (Figure 4A). The structure of the predicted AURKC–IκBα complex indicated that the activation loop of AURKC and ankyrin repeat domain of IκBα are crucial for the interaction. Specifically, the model shows that His164 and Arg165 of AURKC closely contact Val 97 and Lys 98 of IκBα (Figure 4A). To confirm this idea, we constructed a plasmid expressing AURKC protein with mutations at His164, and subjected the protein to co-IP experiments. HEK293T cells were cotransfected with GFP-tagged wild-type AURKC or AURKC H164Y, along with FLAG-tagged IκBα. Whole-cell lysates were subjected to immunoprecipitation with GFP antibody or normal IgG, followed by Western blot analysis with anti-FLAG antibody. IκBα was efficiently co-precipitated with wild-type AURKC, but the AURKC H164Y mutation disrupted the interaction, as expected (Figure 4B). This result demonstrated that His164 of AURKC is important for the interaction with IκBα ankyrin repeat domains I/II/III.

### A small-molecule PPI inhibitor that targets the AURKC–IκBα interaction

Using docking-based virtual screening, we searched for an inhibitor of the AURKC–IκBα binding interface. For this purpose, we used the ChemBridge compound library (http://www.chembridge.com), a commercially available database containing 728,435 molecules. The initial virtual screen for compounds capable of fitting the AURKC–IκBα binding pocket was performed using the HTVS and SP scoring functions of Glide (http://www.schrodinger.com). A total of 35 compounds were selected for further testing.

**Figure 5:**
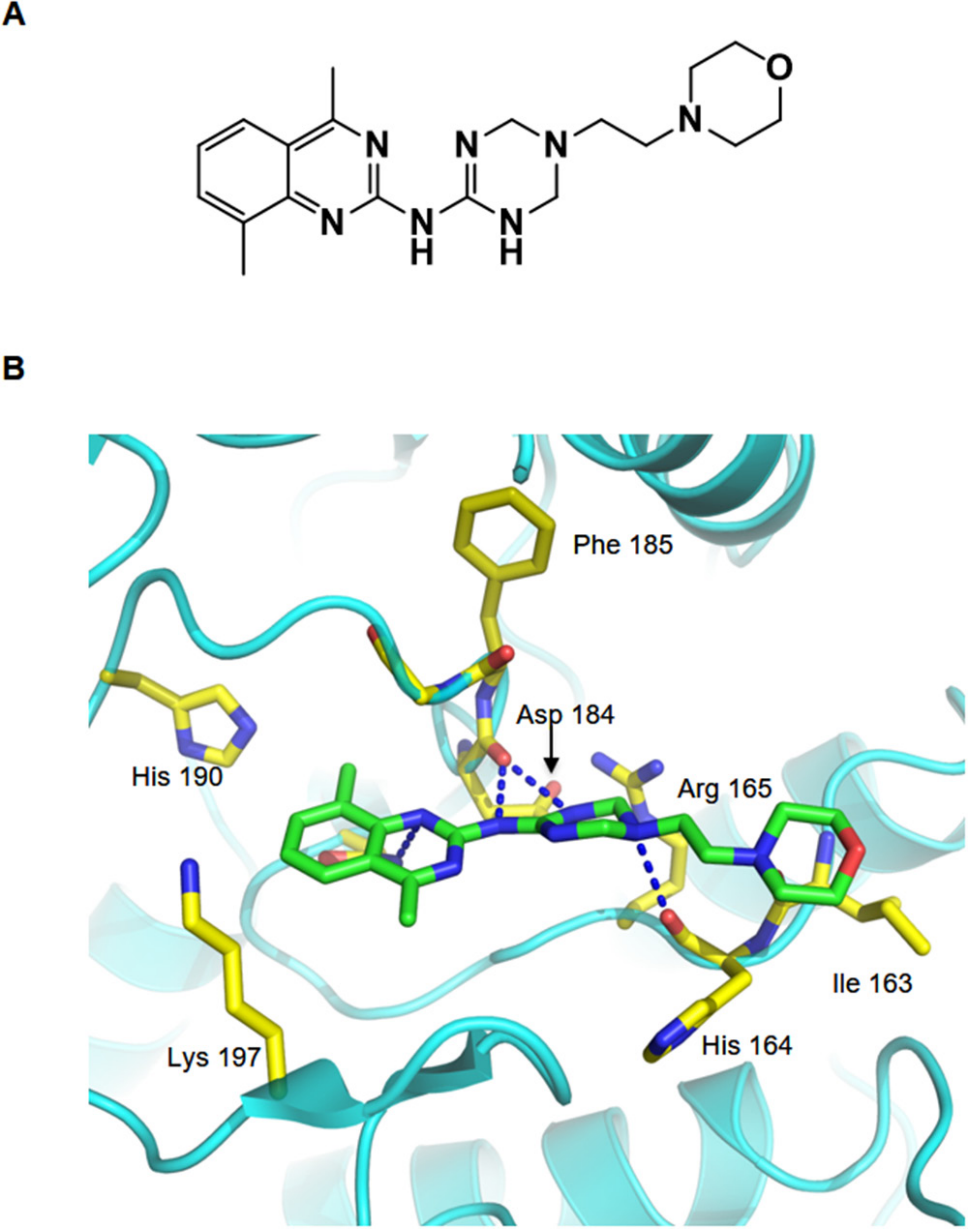
Binding model of AURKC and the IκBα interaction inhibitor (AKCI) **(A)** Structure of the small-molecule PPI inhibitor AKCI. **(B)** Proposed binding model of AURKC and AKCI. Dashed blue lines show hydrogen-bonding interactions between AKCI and the backbone of His164 and Phe185 and the side chain of Asp184.

**Figure 6:**
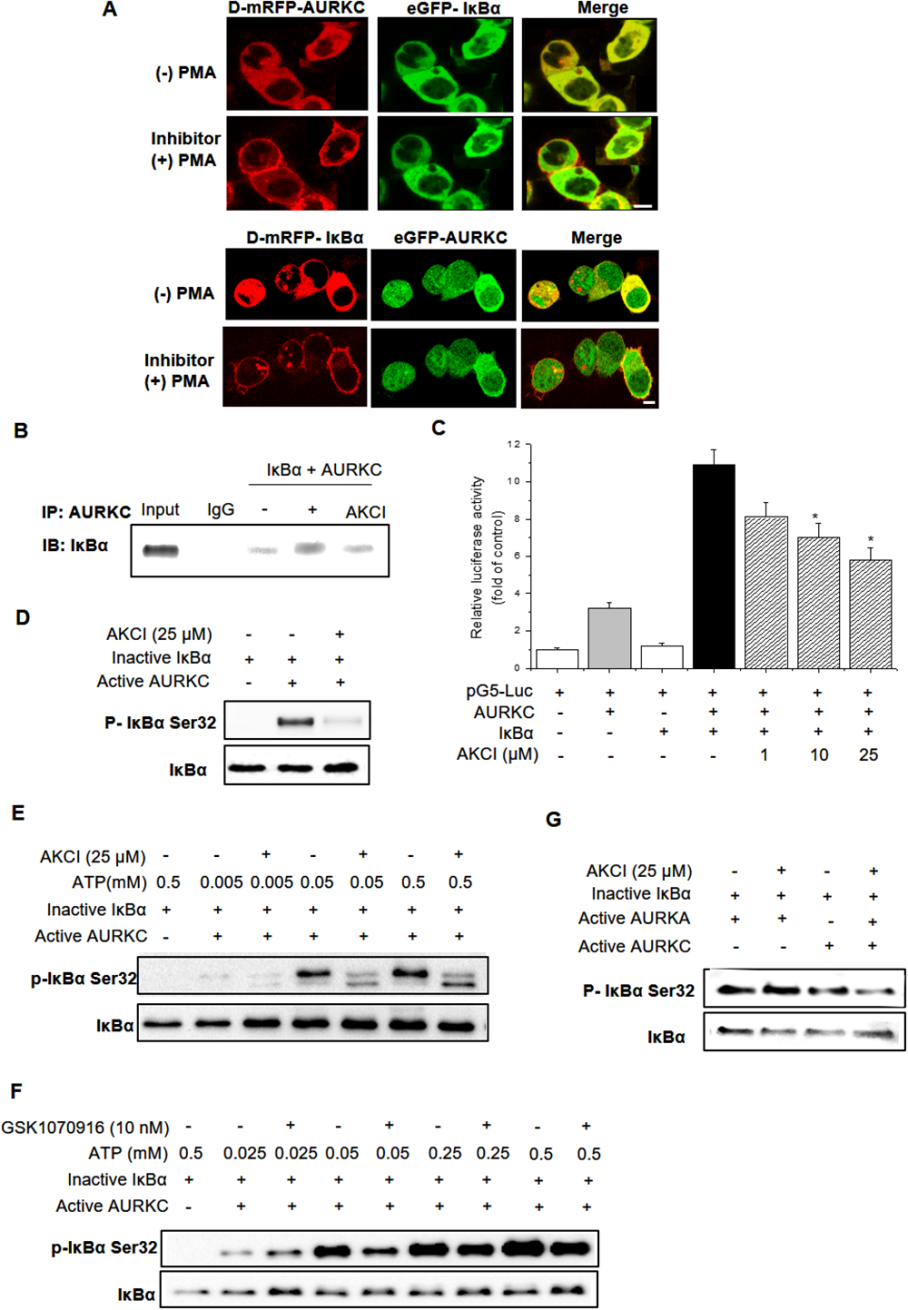
Effects of the small-molecule PPI inhibitor AKCI on the AURKC–IκBα interaction **(A)** HEK293T cells were cotransfected with PKCδ–mRFP–AURKC or IκBα (bait) and eGFP–IκBα or AURKC (prey). Cells were treated with AKCI (25 μM) for 30 min, and then with PMA (300 nM), causing RFP-tagged protein to translocate to the membrane while GFP-tagged protein remained localized in the cytoplasm. Scale bar, 10 μm. **(B)** Cells were treated with AKCI (25 μM) for 24 h. Whole-cell lysates of HEK293T cells transfected with AURKC and IκBα were subjected to immunoprecipitation with either IgG (negative control) or anti-AURKC antibodies, followed by Western blotting of the immunoprecipitates with anti-IκBα antibodies. **(C)** For M2H assays, cells were transfected with pM-BD-pAURKC and pVP-IκBα, and then with AKCI (1–25 μM). Luciferase activity is indicated as the change in relative luminescence units normalized against a negative control. * P < 0.01, significantly different from control as determined by analysis of variance (Newman–Keuls test). **(D)** Inactive IκBα and active AURKC protein were incubated with AKCI (25 μM), and then the mixtures were resolved by SDS-PAGE and subjected to Western blotting using IκBα S32 phospho-specific antibody. **(E, F)** The inhibitory effect of AKCI on IκBα kinase activity is independent of ATP. Inactive IκBα and active AURKC protein were incubated with AKCI (25 μM) or GSK1070916 (10 nM) and various concentrations of ATP, and then the protein mixtures were subjected to Western blotting using IκBα S32 antibody. **(G)** Inactive IκBα and active AURKA protein were incubated with AKCI (25 μM), and then the mixtures were resolved by SDS-PAGE and subjected to Western blotting using an IκBα S32 antibody.

To test the activity of these compounds, we performed cellular protein translocation–based screen assays at non-cytotoxic doses, and identified one compound (AKCI, Figure 5A) that interrupted AURKC–IκBα binding with a half-maximal inhibitory concentration (IC_50_) of 24.9 μM (The IC_50_ values were calculated from the M2H assay results). To further characterize the interactions between AKCI and AURKC, we performed a molecular docking study in which AKCI was docked into the AURKC–IκBα binding site using the Glide docking tool. As shown in Figure 5B, the 1, 3, 5-triazinyl of AKCI was predicted to form hydrogen-bonding interactions with Asp184 and His164 of AURKC. In addition, a morpholino-ethyl group of AKCI was predicted to interact via hydrogen bonding with the Ile163 backbone of AURKC. These results provide support for the interaction between the activation loop of AURKC and ankyrin repeat domain of IκBα. The inhibitory effect of AKCI was evaluated in cellular protein translocation images in HEK293T cells. Upon PMA treatment, the bait (RFP-tagged AURKC or IκBα) was translocated to the cellular membrane (Figure 6A). When bait and prey (GFP-tagged IκBα or AURKC) interact, the prey protein should also migrate to the membrane. However, migration did not occur in the presence of AKCI, suggesting that this compound disrupted the interaction (Figure 6A). The inhibitory effect of AKCI was confirmed using the same technique in CHO-K1 cells (Supplementary Figure 3). To confirm that AKCI inhibits binding between AURKC and IκBα, we performed co-IP and M2H assays. Lysates from HEK293T cells overexpressing full-length AURKC and IκBα, with or without AKCI, were subjected to immunoprecipitation with anti-AURKC antibody and Western blot analysis with anti-IκBα. As shown in Figure 6B, AURKC was efficiently co-precipitated with IκBα in the absence, but not the presence, of AKCI. M2H assays were conducted using plasmids pGC-luc, Bind-AURKC, and Act-IκBα. At concentrations of 10 and 26 μM, AKCI decreased luciferase activity (Figure 6C), confirming that it blocked the AURKC–IκBα interaction. Moreover, AKCI inhibited recombinant AURKC-induced phosphorylation of IκBα (Figure 6D).

Subsequent studies revealed that AKCI, which was identified using a docking-based virtual screening protocol targeting the AURKC–IκBα interaction, does not target the ATP-binding site of the kinase. For this purpose, we performed an ATP-competitive binding assay using AKCI and GSK1070916. The kinase-inhibitory activity of AKCI on AURKC was independent of the ATP concentration, whereas the inhibitory activity of GSK1070916 was inversely proportional to the concentration of ATP (Figure 6E and 6F). Therefore, unlike GSK1070916, AKCI inhibits the AURKC–IκBα interaction by interfering with protein–protein binding, rather than competitively inhibiting ATP binding.

To confirm the specificity of AKCI for AURKC, we performed *in vitro* kinase assays using recombinant AURKA, which is known to interact with IκBα [21]. As shown in Figure 6G, activation of IκBα by AURKA was not altered by AKCI treatment. Thus, AKCI is a specific inhibitor of the interaction between AURKC and IκBα.

### AKCI exerts an anticancer effect and induces G2/M arrest in MDA-MB-231 breast cancer cells

**Figure 7:**
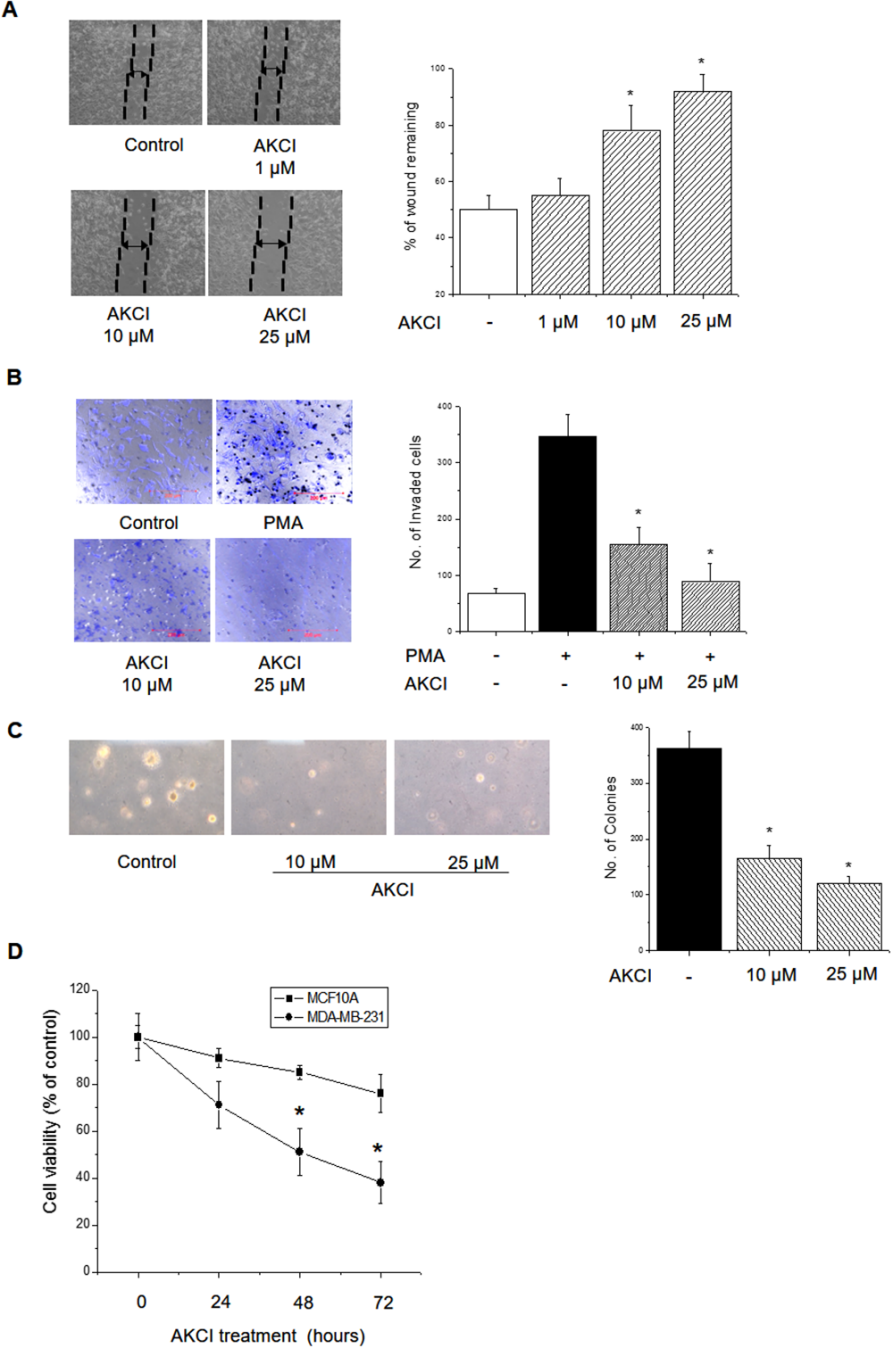

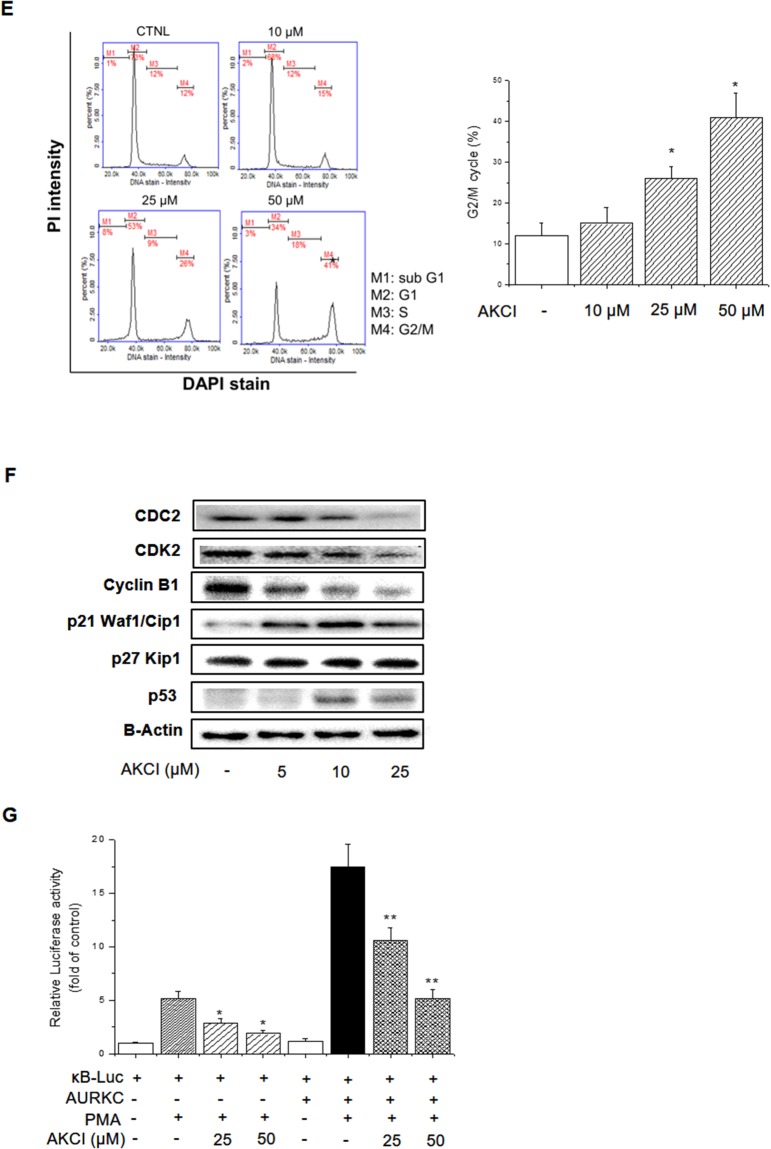
Inhibitory effects of AKCI on cancer cell transformation in breast cancer cells **(A)** AKCI inhibits migration by MDA-MB-231. Cells were seeded into culture inserts and incubated overnight. Cell proliferation was stopped by mitomycin C treatment for 2 h, and culture inserts were removed to introduce a cell-free gap. The cells were treated with various concentrations of AKCI for 24 h, and cell migration was observed under a microscope. **(B)** AKCI inhibits invasion by MDA-MB-231. Cells were seeded into the inserts of Boyden chambers and incubated overnight. The cells were treated with the indicated doses of AKCI, and cell invasion was allowed to proceed for 24 h. The percentage of invasive MDA-MB-231 cells was significantly reduced following AKCI treatment (*p < 0.05). **(C)** MDA-MB-231 cells (1 × 10^3^ cells/ml) were mixed with 0.3% soft agar and grown on a 0.6% agarose base layer. Anchorage-independent colony formation was diminished by AKCI. **(D)** Cell viability of AKCI-treated cells. Normal MCF10A and malignant MDA-MB-231 cells were treated with 25 μM AKCI for the indicated times (days), and viability was determined using the CCK8 kit. MDA-MB-231 cells were more sensitive to AKCI-mediated cell growth inhibition than MCF10A cells. * P < 0.01, significantly different from control. **(E)** Effects of AKCI on cell-cycle distribution in MDA-MB-231 cells. Representative plots were presented as a result * P < 0.01, significantly different from control G2/M cycle. **(F)** Effect of AKCI on expression levels of CDK1/CDC2, CDK2, cyclin B1, p21^Waf1/Cip1^, p27 Kip1, and p53 in MDA-MB-231 cells. Cells were treated with AKCI at 5, 10, and 25 μM for 24 h. Actin was used as the internal control. **(G)** Inhibitory effects of AKCI on transcriptional activity of NF-κB. Cells were cotransfected with pGL3-NF-κB-luc, pcDNA3.1-AURKC, and pCMV-β-gal, and then treated with AKCI (10 and 25 μM) for 24 h, followed by determination of luciferase activity. * P < 0.01 and ** P < 0.01, significantly different from PMA treatment in control and AURKC-overexpressing cells.

To determine whether AKCI exerts an anticancer effect by targeting AURKC protein in MDA-MB-231 breast cancer cells, we performed migration, invasion, and colony formation assays. As shown in Figure 7A, AKCI markedly decreased the migration of highly invasive MDA-MB-231 cells in a concentration-dependent manner. In addition, the inhibitor significantly decreased the number of invading cells following treatment with PMA (Figure 7B). Likewise, AKCI treatment diminished colony formation (Figure 7C). Breast tumors express significantly higher levels of AURKC than normal breast tissues, and the *AURKC* gene is amplified in MDA-MB-231 cells [22]. The protein affects cell division via its serine/threonine kinase activity and its capacity to organize microtubules in relation to centrosome/spindle function during mitosis [13]. Western blot analysis revealed high levels of both total and phosphorylated AURKC in MDA-MB-231 cells, in comparison with the nonmalignant cell line MCF10A. By contrast, endogenous IκBα protein expression did not differ between these cell types (Supplementary Figure 5), although the IκBα phosphorylation level was higher in MDA-MB-231 than in MCF10A cells (Supplementary Figure 5).

To determine whether the anti-proliferative effect of AKCI is cancer cell–specific, we treated normal and cancer cells with the drug and measured proliferation using the CCK8 kit. MDA-MB-231 cells exhibited reduced proliferation when treated with 25 μM AKCI for 24, 48, and 72 h (Figure 7D). By contrast, MCF10A cells (non-tumorigenic) exhibited no significant decrease in proliferation under the same conditions. Thus, cancer cells are more sensitive than non-tumorigenic cells to the anti-proliferative effects of AKCI.

In eukaryotes, cellular proliferation is controlled primarily by regulation of the cell cycle, which consists of four distinct sequential phases (G0/G1, S, G2, and M) [23] governed by cyclins, CDKs, and cyclin-dependent kinases. In particular, cyclin B and CDK1 proteins regulate the progression of G2/M phase [24]. Cells arrest in G2/M phase following DNA damage, and are more susceptible to the cytotoxic effects of radiotherapy at this stage of the cell cycle [25]. Induction of G2/M phase arrest promotes cell death, a useful strategy in cancer therapeutics [26]. To determine whether AKCI modulates the cell cycle in malignant breast cancer cells, we assessed the cell-cycle distribution of MDA-MB-231 cells exposed to AKCI for 24 h. Treatment with AKCI at various concentrations arrested the cells at G2/M transition (Figure 7E). In addition, we examined the effect of AKCI on the expression levels of CDK1/CDC2, CDK2, cyclin B1, p21^Waf1/Cip1^, p27 Kip1, and p53 in MDA-MB-231 cells. The Cip/Kip family, including p21^Waf1/Cip1^ and p27 Kip1, binds to cyclin–CDK complexes and prevents kinase activation, subsequently blocking cell-cycle progression at G2/M phase [27]. p53 is a transcription factor that upregulates a number of important cell cycle–modulating genes, including p21^Waf1/Cip1^, which binds to the CDK1/CDC2–cyclin B1 complex to induce cell-cycle arrest [7]. In MDA-MB-231 cells, the level of CDK1/CDC2 was remarkably reduced following AKCI treatment, whereas the levels of p21^Waf1/Cip1^ and p53 were markedly elevated (Figure 7F). These results suggested that AKCI, which targets AURKC, might diminish cellular transformation by inducing G2/M arrest in MDA-MB-231 breast cancer cells.

Additionally, AKCI decreased PMA-induced NF-κB promoter activity in both wild-type and AURKC-overexpressing cells (Figure 7G). Therefore, AURKC overexpression might lead indirectly to NF-κB activation, mediated by the interaction between AURKC and IκBα. This aberrant activation of NF-κB could be attenuated by AKCI.

## DISCUSSION

In normal cells, the function of AURKC is predominantly restricted to meiosis; however, it is aberrantly expressed in various cancer cell lines, and contributes to oncogenesis [28–30]. AURKC overexpression induces abnormal cell division resulting in centrosome amplification, multinucleation, and increased proliferation [31]. In addition, cells overexpressing AURKC form colony foci on soft agar, and transplantation of these cells induces tumor formation in nude mice. Recently, Zekri et al. reported amplification of the *AURKC* gene in MDA-MB-231 breast cancer cells [22]. However, the role of AURKC in carcinogenesis remains incompletely understood at a mechanistic level. Because AURKC is overexpressed in breast cancer cells, its PPI networks provide valuable information regarding its contribution to oncogenesis.

In this study, with the goal of elucidating the mechanistic role of AURKC in cancer cell transformation, we identified IκBα as a novel AURKC binding partner and an inhibitor capable of targeting the AURKC–IκBα interaction in breast cancer cells. Specifically, we screened for a small-molecule inhibitor that could control the PPI of AURKC by targeting a non-catalytic site in the interface. Such strategies have enabled drug discovery to move beyond the typical enzyme targets, such as kinases, and address disease state–relevant regulatory complexes involved in biological processes such as intracellular signal transduction and transcription. However targeting PPIs poses unique challenges, because the target’s structure (i.e., the interaction site between two proteins) is often unknown, and is usually a relatively large area with physico-chemical properties distinct from those of typical catalytic sites.

We identified the interaction between AURKC and IκBα by a translocation-based cellular system using fluorescence tagging plasmids, co-IP, and M2H assays. Colony-forming assays revealed that IκBα is essential for AURKC-induced transformation in MDA-MB-231 breast cancer cells. *In vitro* kinase assays revealed that AURKC kinase might induce site-specific phosphorylation of IκBα, and furthermore that the IκBα ankyrin repeat domain is important for its interaction with AURKC. Additionally, co-IP assays using an AURKC mutant indicated that His164 and Arg165 of AURKC are important for the interaction with IκBα ankyrin repeat domains I/II/III. In general, activation of NF-κB occurs by release of the transcription factor complex from IκB molecules, or by cleavage of the inhibitory ankyrin repeat domains of p100 and p105 [32]. AURKC directly induces IκBα activation via an interaction between the two proteins, leading to phosphorylation of IκBα. Although AURKC alone could not induce NF-κB activation, NF-κB was activated when AURKC was overexpressed in the presence of the NF-κB inducer PMA.

Tumor cells can acquire elevated NF-κB activity via intrinsic or extrinsic factors [33]. On the one hand, elevated NF-κB activity can be directly induced by mutations of NF-κB genes and/or oncogenes that activate the NF-κB signaling pathway. On the other hand, tumor NF-κB activation can result from high levels of cytokines in the tumor microenvironment [34, 35]. Consistent with this, the NF-κB activator PMA stimulates cytokine secretion.

Our results demonstrate that the AURKC–IκBα interaction induces high levels of NF-κB activation. Moreover, we identified a novel phosphorylation site of IκBα using matrix-assisted laser desorption/ionization (MALDI) mass spectrometry (unpublished data). Hence, we postulate that AURKC-mediated serine phosphorylation of IκBα contributes to various types of cancer cell transformation. We would like to report the role of IκBα in the new phosphorylation site through functional studies. Based on the domain mapping experiment (Figure 3D), we performed computational modeling and predicted a small-molecule PPI inhibitor of the AURKC–IκBα interaction. *In silico* modeling, translocation-based cellular assay using fluorescence tagging plasmids, co-IP, M2H, and *in vitro* kinase assays revealed that the small-molecule inhibitor AKCI inhibited binding between AURKC and IκBα by targeting a non-catalytic site of AURKC. Furthermore, AKCI inhibited migration, invasion, and colony formation by inducing G2/M arrest in MDA-MB-231 cells. However, AKCI had no effect on apoptosis or expression of MMP-9 (Supplementary Figures 6 and 7). Finally, AKCI inhibited PMA-induced NF-kB promoter activity, which plays pivotal roles in cancer cell transformation. Based on our results, the AURKC–IκBα interaction represents a promising therapeutic target for treatment of breast cancer.

## MATERIALS AND METHODS

### Materials

Phorbol myristate acetate (PMA) was obtained from Sigma Chemical Co. (St. Louis, MO, USA), and AKCI (4, 8-dimethyl-N-{5-[2-(4-morpholinyl)ethyl]-1, 4, 5, 6-tetrahydro-1, 3, 5-triazin-2-yl}-2-quinazolinamine) was purchased from ChemBridge Corporation (San Diego, CA, USA). GSK1070916 was obtained from Selleck Chemicals LLC (Suffolk, UK). Fetal bovine serum (FBS), penicillin–streptomycin solution, and trypsin were obtained from Life Technologies, Inc. (Carlsbad, CA, USA). The HaloTag vector system and luciferase assay reagent were purchased from Promega (Madison, WI, USA). pCMV-β-gal and RFP antibody were obtained from Clontech (Palo Alto, CA, USA). The IκB kinase assay kit was obtained from R&D Biosystems (Minneapolis, MN, USA), and the protein assay kit was purchased from Bio-Rad Laboratories, Inc. (Hercules, CA, USA). Activated AURKC and IKKβ were obtained from SignalChem (Richmond, Canada). Antibodies against AURKC and phospho-AURKC were obtained from GeneTex Inc. (San Antonio, TX, USA) and Aviva Systems Biology (San Diego, CA, USA), respectively. Antibodies against IκBα, phospho-IκBα S32, and GFP were obtained from Santa Cruz Biotechnology (Dallas, TX, USA), and anti-phospho-IκBα S36 was purchased from Abcam (Cambridge, MA, USA). Antibodies against phospho-IκBα S32/36, p21, p53, CDC2, and cyclin B1, as well as secondary antibodies, were obtained from Cell Signaling Technology (Beverly, MA, USA). The BioCoat Matrigel Invasion Chamber for invasion assay was obtained from BD Sciences (San Jose, CA, USA). TurboFect was obtained from Thermo Fisher Scientific (Waltham, MA, USA). The CCK8 kit was obtained from Dojindo Laboratories (Kumamoto, Japan).

### Cell culture and treatment

The normal breast cell line MCF10A and MDA-MB-231 breast cancer cells were obtained from the American Type Culture Collection (Rockville, MD, USA). HEK293T cells were obtained from the Korean Cell Line Bank (Seoul, South Korea). All cells were cultured in Dulbecco’s modified Eagle’s medium (DMEM), high glucose, supplemented with 10% FBS, in a humidified 5% CO_2_ incubator at 37°C. Stock solutions of PMA and A/I inhibitor (AKCI) were prepared in dimethylsulfoxide (DMSO) and added directly to culture media. Control cells were treated only with DMSO, and the final DMSO concentration was always <0.2%.

### Molecular cloning and transfection of HEK293T cells

The C-terminal fusion constructs PKCδ/monomeric red fluorescent protein (mRFP)/AURKC and /IκBα (bait) were generated using the EcoRI and BamHI restriction site in C-terminal fusion constructs PKCδ/monomeric red fluorescent protein (mRFP) [17]. For analysis of PPI, the cDNAs of 520 human kinases were amplified by PCR and introduced into the mammalian expression plasmid pEGFP-C3. Human *AURKC* cDNA was subcloned into the *Eco*RI and *Bam*HI sites of vector pcDNA3.1. The following primers were used for molecular cloning: PKCδ-mRFP/pEGFP and pCDNA3.1-AURKC (forward) 5’-gctgaattctgatgcggcgcctcacagtcg-3’; PKCδ-mRFP/pEGFP, PKCδ-mRFP/pEGFP, and pCDNA3.1-AURKC (reverse) 5’-gctggatcctaggaagccatctgagcacag-3’; PKCδ-mRFP/pEGFP-IκBα (forward) 5’-gaagaattctgatgttccaggcggccgagcg-3’; and PKCδ-mRFP/pEGFP-IκBα (reverse) 5’-gaaggattccgtcataaacgtcagacgctggcctccaa-3’. All positive clones containing cDNA inserts were confirmed by restriction enzyme mapping and DNA sequence analysis (Cosmo Genetech, Daejeon, South Korea).

### Analysis of PPI and small-molecule inhibitor targeting of PPI by laser scanning fluorescence microscopy imaging

HEK293T cells were cotransfected with PKCδ–mRFP–AURKC (bait) and eGFP-prey protein (520 kinases, including IκBα). Fluorescence images were acquired on a confocal laser scanning fluorescence microscope (LSM710, Carl Zeiss) equipped with a 40× objective. Images were analyzed using the ZEN2009 software (Carl Zeiss).

### Co-immunoprecipitation (co-IP)

HEK293T cells were cotransfected with pcDNA3.1-AURKC myc-his and pCMV6-IkB myc-Flag using TurboFect (Thermo Fisher Scientific). Twenty-four hours after transfection, cells were washed with 1× PBS and lysed with 1 ml of IP lysis buffer (50 mM Tris pH 7.4, 150 mM NaCl, 1 mM EDTA, 0.5% NP-40, 5% glycerol) supplemented with protease inhibitor cocktail mix (Roche). Cell lysates were pre-cleared by pre-incubation with protein A–Sepharose 4 Fast Flow beads (GE Healthcare) for 30 min, and then incubated for 2 h with fresh beads and 1:200 dilutions of anti-IκBα, anti-AURKC, and anti-goat IgG antibodies. The beads were washed once with IP lysis buffer and twice with PBS, and the immune complexes were released from the beads by boiling in sample buffer for 5 min. Following 10% SDS-PAGE, immunoprecipitates were transferred onto polyvinylidene difluoride (PVDF) membrane and immunoblotted using the indicated antibodies. Proteins were visualized using the enhanced chemiluminescence (ECL) detection system (GE Healthcare).

### Mammalian two-hybrid assay

For transfections, equal amounts of plasmids pBIND-AURKC, pACT-IκBα, and pG5-luciferase were combined; the total amount of DNA was no more than 100 ng/well. Transfections were performed with TurboFect. Cells were disrupted by addition of 200 μl of cell lysis buffer directly into each well of the 48-well plate, and then aliquots of 70 μl were added to individual wells of a 96-well luminescence plate. Luminescence activity was measured automatically on a SpectraMax M5 microplate reader (Molecular Devices). Relative luciferase activity was calculated by normalization against the pG5-luciferase basal control. *Renilla* luciferase activity assay was used for assessment of transfection efficiency and protein level.

### *In situ* proximity ligation assay (PLA)

Cells were seeded onto 24-well plates with glass coverslips at the bottom of each well. After transfection of target vectors, cells were fixed in 4% paraformaldehyde for 5 min, and then washed four times with PBS. Cover glasses were attached to glass slides using Canada Balsam (Sigma Chemical Co, St. Louis, MO, USA) to facilitate the manipulations in the next step. All steps of the Duo-Link (Promega, Madison, WI, USA) protocol were performed as open-droplet reactions on the cover glass to ensure complete coverage of the cells. Primary antibodies against AURKC and IκBα were diluted 1:100 in Duo-Link antibody diluent solution, applied to the cover glass. The next day, the slides were washed three times in Duo-Link wash buffer A to remove excess antibody. After washing, antibody–oligonucleotide conjugates were applied to cells for 2 h and ligated for 30 min at 37°C in Duo-Link ligation mixture. Subsequently, ligated templates were amplified in Duo-Link amplification mixture for 100 min at 37°C. After the reaction, the samples were washed twice for 10 min in Duo-Link wash buffer B and once for 1 min in 0.1× wash buffer B, and then mounted with ProLong Diamond Antifade Mountant with DAPI (Life Technologies, Carlsbad, CA, USA). Image acquisition was performed on a Zeiss 710 confocal microscope under 4× magnification.

### Cell-based ELISA of IκBα

IκBα activation was examined using a cell-based ELISA phospho-IκBα (S32/S36) assay kit (R&D Biosystems). Briefly, HEK293T cells were seeded in black 96-well plates, and then transfected with AURKC expression vector and shRNA (CCACGATAATAGAGGAGTTGGCAGATGCC) for 24 h. Subsequently, the cells were fixed and incubated with primary and secondary antibodies. After addition of substrate, fluorescence was detected on a SpectraMax M5 microplate reader.

### Purification of IκBα protein using the HaloTag system

Full-length human IκBα was cloned into vector pFN18A (Promega), and the resultant plasmid was transformed into KRX cells (Promega) grown in LB media (+ampicillin) supplemented with glucose and rhamnose to induce expression without isopropyl-β-D-thiogalactoside (IPTG). After the cells reached an OD_600_ of 0.5–0.6 at 37°C, the temperature was reduced to 20°C for overnight expression. For IκBα purification, cell pellets were resuspended in 5 ml of HaloTag purification buffer (50 mM HEPES [pH 7.5] and 150 mM NaCl) supplemented with 1× Protease Inhibitor (Thermo Fisher Scientific), and then sonicated on ice using a Vibra cell sonicator (5 min total on time; 1 min on/1 min off; amplitude = 40–60, pulse = 4). Lysates were centrifuged at 10,000 *g* for 30 min at 4°C, and the supernatants were directly applied to pre-equilibrated HaloLink resin (Promega). Binding to the resin was conducted at room temperature for 1 h with constant end-over-end rotation, followed by three washes in 10 ml of purification buffer for 5 min each. Target proteins were released from the resin by proteolytic cleavage using a Halo TEV enzyme for 1 h at room temperature. Supernatants containing the released protein of interest and TEV protease were carefully transferred to a new tube. To remove TEV protease, HisLink resin was added to the tube and allowed to bind at room temperature for 20 min with constant end-over-end rotation. After centrifugation at 1,000 *g* for 5 min, the supernatant was transferred to another tube. To determine purity, purified proteins were resolved by SDS-PAGE, and gels were stained with Coomassie Brilliant Blue (Thermo Fisher Scientific).

### Immunoblotting and kinase assays

The IκBα full-length (1–317 aa) and deletion constructs (1–175, 1–277, and 1–72/278–317 aa) were amplified by PCR and cloned into the HaloTag pFN18A plasmid. The IκBα S32A, S36A, and S32/36A, and AURKC H164Y, mutations were introduced using the QuikChange Lightning site-directed mutagenesis kit (Stratagene, Santa Clara, CA, USA). Recombinant active AURKC (100 ng) protein and substrate IκBα or IκBα mutant (S32A, S36A, S32/36A, 1–175 aa, 1–277 aa, or 1–72/278–317 aa) were incubated in the presence or absence of AKCI for 10 min at 30°C. The mixture was suspended in kinase buffer supplemented with 10 μl of diluted ATP solution. Protein complexes were resolved by 10% SDS-PAGE, followed by electroblotting onto PVDF membranes. The membranes were probed with the appropriate primary antibodies, followed by incubation with horseradish peroxidase–conjugated secondary antibody. The blots were visualized using the ECL Western blot kit.

### Establishment of AURKC-overexpressing and -knockdown cell lines

Viral supernatants were applied to MDA-MB-231 cells in 6-well plates overnight in the presence of LentiBoost (Sirion Biotech GmbH, Martinsried, Germany). After infection, MDA-MB-231 cells were selected for 48 h with 2 μg/ml puromycin, and then sorted by GFP intensity on a MoFlo Astrios flow cytometer (Beckman Coulter, USA). Validation of GFP-positive cells was performed by confocal microscopy (LSM710, Carl Zeiss).

### Binding model prediction of AURKC and IκBα

The model of IκBα–AURKC binding was generated using the ZDOCK tool in Discovery Studio 4.5 (BIOVIA). The structure of IκBα was obtained from the Protein Data Bank (PDB code: 1ikn). The homology model of the AURKC structure was constructed using MODELLER in Discovery Studio4.5, using the structure of AURKA (PDB code: 2j4z) as a template. The initial IκBα and AURKC structures were minimized by the steepest-descent method for 5,000 iterations using the CHARMm force field. The AURKC–IκBα docking model were generated top 2,000 predictions for each case and it was generated using ZDOCK at a 6° rotational sampling density with each of the scoring functions. Finally, the top-scoring cluster was selected as the model for the AURKC–IκBα complex.

### Docking model prediction of AURKC–IκBα interaction inhibitor

To generate a binding model of the AURKC–IκBα interaction inhibitor, the homology model structure of AURKC was subjected to *in silico* docking with AKCI. The protein structure was prepared according to the standard procedure for the Protein Preparation Wizard in Schrödinger suite 2015-2. After complete preparation of ligands and protein for docking, receptor-grid files were generated. Ligand docking into the AURKC–IκBα binding interface site was carried out using the Schrödinger docking program, Glide. The energy-minimized AURKC–IκBα interaction inhibitor was docked into the prepared receptor grid. The best-docked pose was selected based on the lowest Glide score. The molecular graphics for the inhibitor-binding pocket and refined docking model for the AKCI were generated using PyMol (http://www.pymol.org).

### Virtual screening

To identify compounds that inhibit the AURKC–IκBα interaction by binding the PPI, a docking-based virtual screen of the AURKC–IκBα binding interface was performed using Glide. The HTVS (High Throughput Virtual Screening) and SP (Standard Precision) scoring functions were used, granting full flexibility to the ligands. The top-ranking SP poses of each compound were selected. The compound dataset used for the virtual screening experiments was a commercially available chemical library (http://www.chembridge.com, 728,435 molecules).

### Assay for proliferative activity

Cell cytotoxicity was monitored using a Cell Counting Kit-8 (CCK8, Dojindo Laboratories). Briefly, MDA-MB-231 cells in DMEM, high glucose, containing 10% FBS were seeded into 96-well plates. AKCI (25 μM) was added to the wells, and the plates were incubated at 37°C for 24, 48, or 72 h. CCK8 solution was added (10 μl per well), and the plates were incubated for 1–4 h at 37°C. Absorbance was detected at 450 nm with a SpectraMax M5 microplate reader. All experiments were repeated three times independently.

### Cell migration and invasion assay

MDA-MB-231 cells were seeded into 24-well plates and cultured overnight. The cells were treated with mitomycin C (10 μg/ml) for 2 h, and the culture inserts were removed to open a cell-free gap. The cells were treated with the indicated doses of AKCI or PMA for 24 h, and cell migration was observed under a light microscope. The migrated area was measured using the ImageJ software (v. 1.45). The effect of A/I inhibitor on cancer cell invasion was measured using a Matrigel-coated invasion chamber (Corning Incorporated, Corning, NY, USA). Briefly, 1 × 10^4^ MDA-MB-231 cells were seeded into an insert chamber with FBS-free media, supplemented with the indicated doses of A/I inhibitor (AKCI), and cultured in 24-well plates supplemented with complete media for the indicated times. The cells were fixed with 4% formaldehyde, permeabilized with methanol, and stained with crystal violet. The stained cells were observed under a light microscope, and those that had migrated were counted.

### Soft agar assay

For the soft agar assay, cells were trypsinized to generate single-cell suspensions and counted. 1.2% agarose (Promega, Madison, WI, USA) dissolved in medium was plated on the bottom of each well. Single-cell suspensions (1,000 cells per well) were mixed with 0.6% agarose and seeded on top of the bottom agar. All assays were performed in triplicate. The cells were incubated at 37°C for 6 weeks to allow colony formation. Images were captured using a Zeiss inverted wide-field microscope equipped with a Canon G12 camera.

### Colony formation assay

Cells were seeded in 6-well plates at 1,000 cells/well and cultured for 8 days to allow colony formation. After incubation, cells were washed with PBS, stained with 0.1% crystal violet (AMRESCO, Solon, OH, USA) in 50% methanol, and counted.

### Cell-cycle analysis

Cells were treated with AKCI and then trypsinized, washed with PBS, and subjected to cell-cycle analysis on a NucleoCounter NC-3000 (ChemoMetec, Allerød, Denmark). Two-step analysis was performed in which the cells were lysed and the nuclei were stained with DAPI [18].

### Transient transfection and the NF-κB luciferase assay

HEK293T cells were transiently transfected with the NF-κB promoter–luciferase construct and pcDNA3.1-AURKC using the TurboFect reagent. After overnight transfection, cells were incubated with PMA or AKCI for 24 h, and whole-cell lysates were prepared. Whole-cell extract (70 μl) was mixed with 70 μl of the luciferase assay reagent and analyzed on a SpectraMax M5 luminometer.

### Statistical analysis

All experiments were repeated at least three times. One-way analysis of variance (ANOVA) was used to determine the significance of differences between treatment groups. The Newman–Keuls test was used for multi-group comparisons. Statistical significance was defined as *p* < 0.01.

## SUPPLEMENTARY MATERIALS FIGURES


