# Supplementary material for: A small-molecule inhibitor targeting the AURKC–IκBα interaction decreases transformed growth of MDA-MB-231 breast cancer cells
Source: Oncotarget. 2017 Jun 29;8(41):69691–708. doi: 10.18632/oncotarget.18883 (PMC5642509; doi:10.18632/oncotarget.18883)
Supplement: Supplementary file 1 [file oncotarget-08-69691-s001.pdf]

A small-molecule inhibitor targeting the AURKC–IκBα interaction decreases transformed growth of MDA-MB-231 breast cancer cells

SUPPLEMENTARY MATERIALS

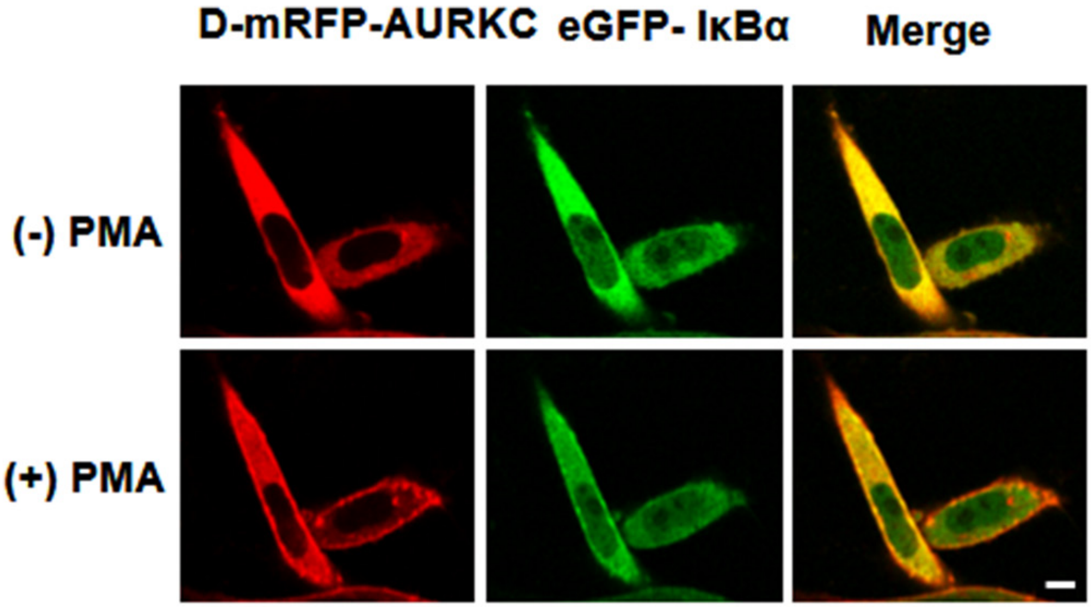

Supplementary Figure 1: Confirmation of AURKC–IκBα interaction in CHO-K1 cells. Cells were cotransfected with PKCδ–mRFP–AURKC (bait) and eGFP–IκBα (prey). Before PMA treatment, the mRFP-tagged bait and GFP-tagged prey proteins were localized in the cytoplasm. However, after PMA (300 nM) was added, bait and prey proteins were relocalized to the plasma membrane due to the translocation properties of PKCδ, as in HEK293T cells. Scale bar, 10 µm.

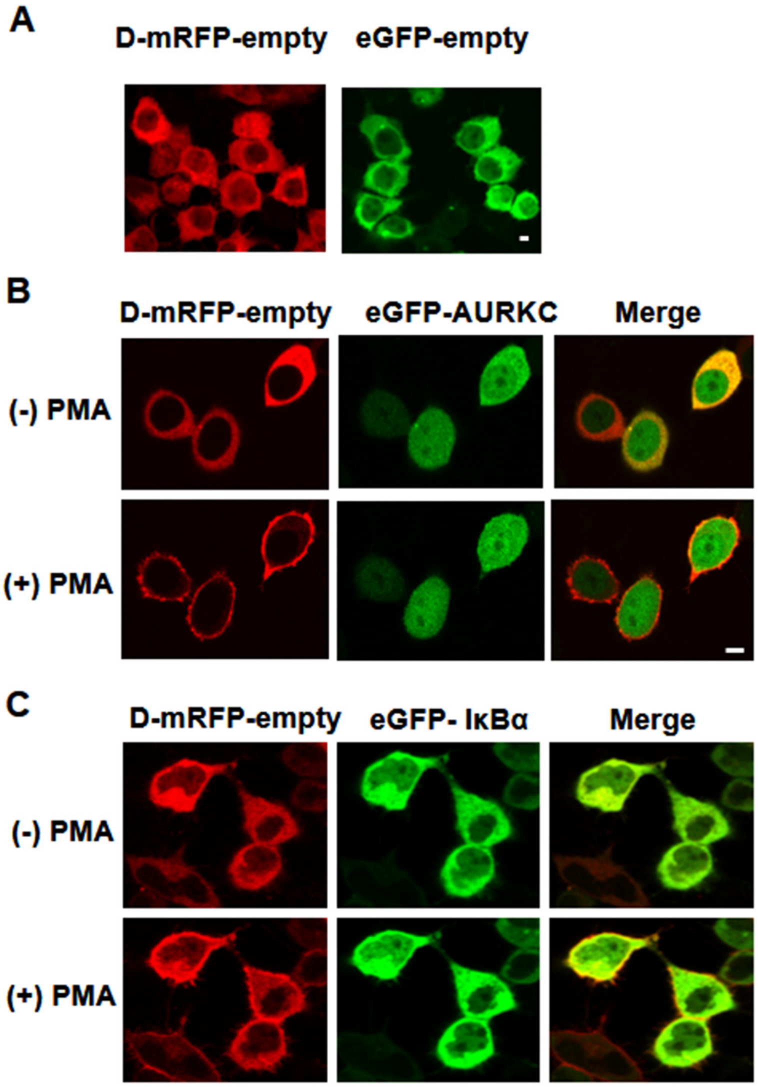

Supplementary Figure 2: Images of empty vector and negative control bait/target protein pairs in cells. (A) Cells were cotransfected with PKCδ–mRFP and eGFP empty vectors. Following PMA treatment, only the RFP-tagged bait protein was translocated to the cell membrane, whereas GFP remained in the cytoplasm. **(B)** mRFP and eGFP–AURKC. **(C)** mRFP and eGFP–IκBα. Scale bar, 10 µm.

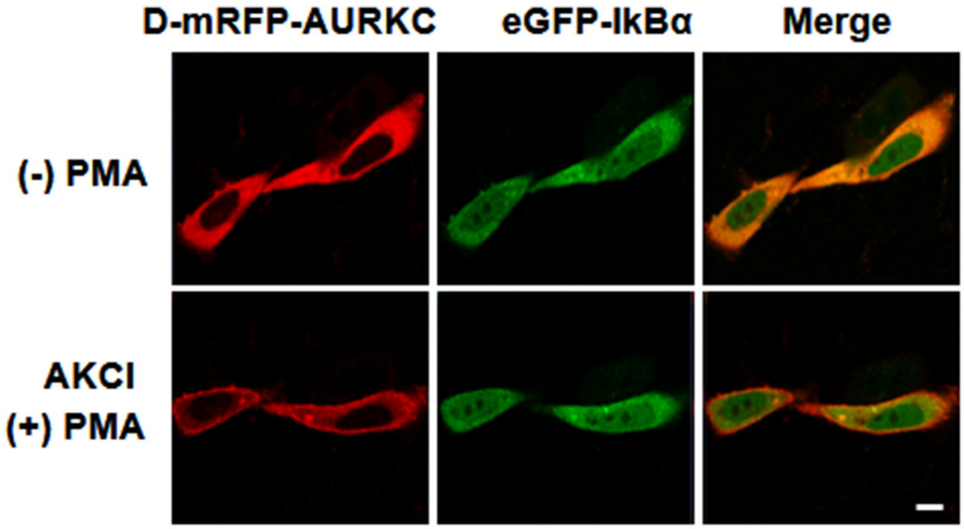

Supplementary Figure 3: Inhibitory effect of AKCI on the AURKC–IκBα interaction in CHO-K1 cells. Cells were cotransfected with PKCδ–mRFP–AURKC (bait) and eGFP–IκBα (prey), treated with AKCI (25 μM) for 30 min, and then treated with PMA (300 nM). RFP-tagged protein was translocated to the membrane, whereas GFP-tagged protein remained in the cytoplasm. Scale bar, 10 μm.

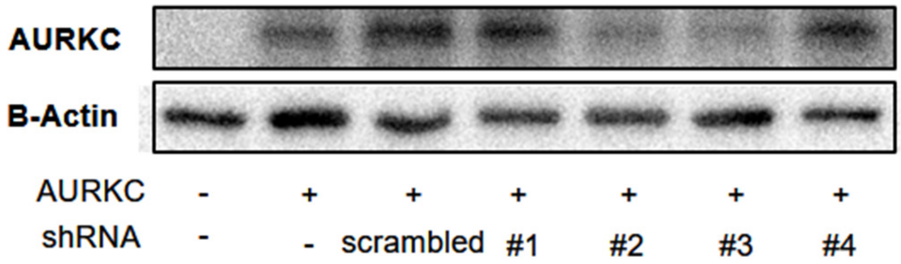

Supplementary Figure 4: Effects of shRNA of AURKC in HEK293T cells. Immunoblot analysis of HEK293T cells 48 h after transfection with shRNAs targeting AURKC or a scrambled control shRNA.

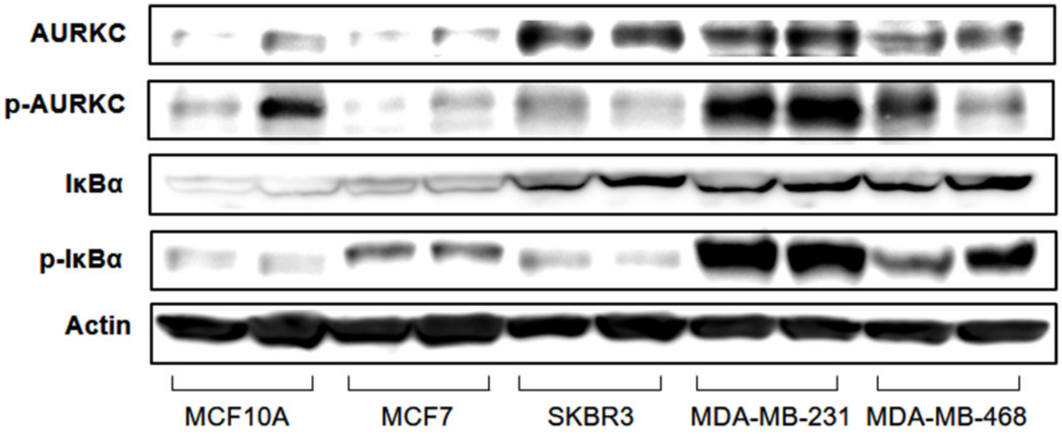

Supplementary Figure 5: Endogenous expression of AURKC and IκBα in breast cell lines. Immunoblot analysis of AURKC and IκBα protein expression and phosphorylation in a panel of breast cancer cell lines.

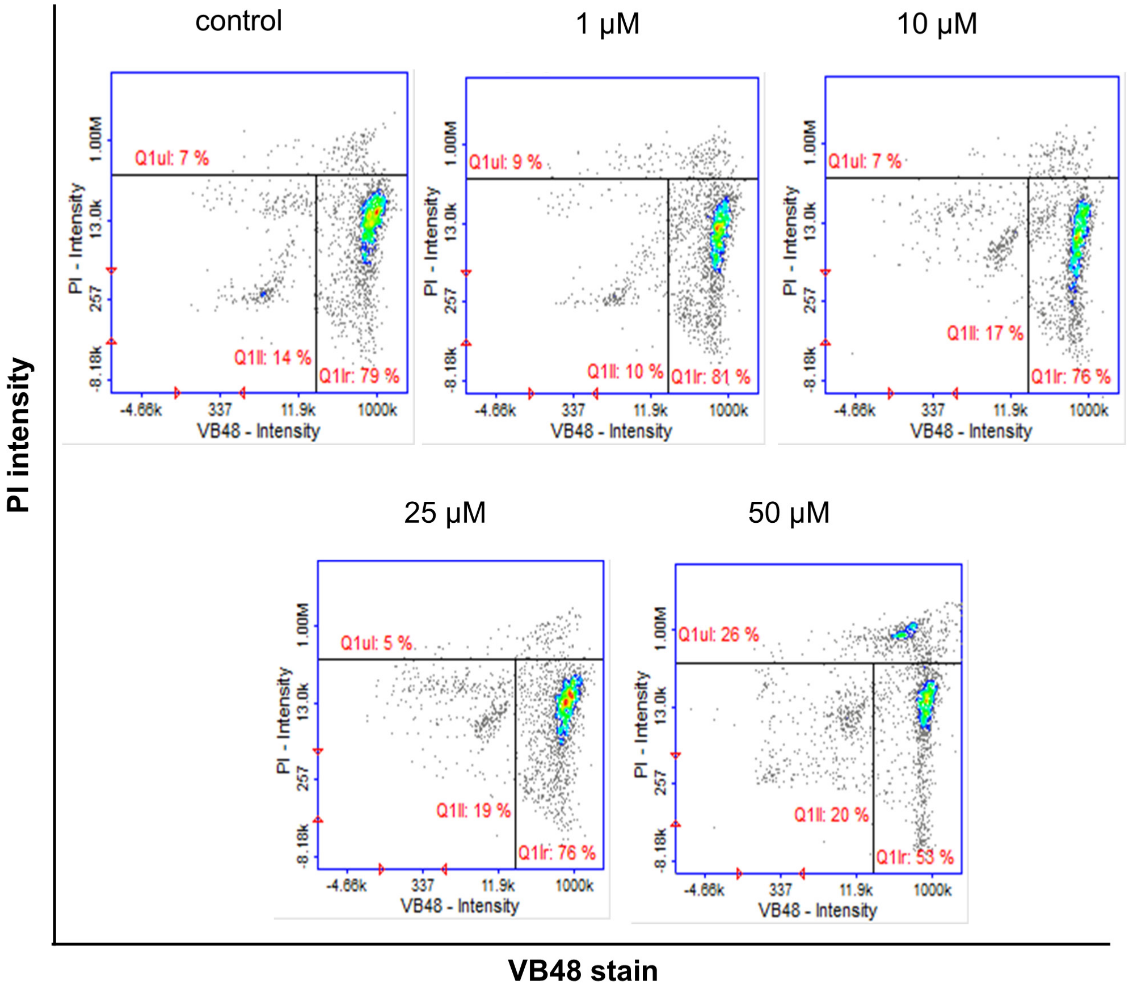

Supplementary Figure 6: Effects of AKCI on apoptosis in MDA-MB-231 cells. Representative plots were presented as a result of repeated experiments.

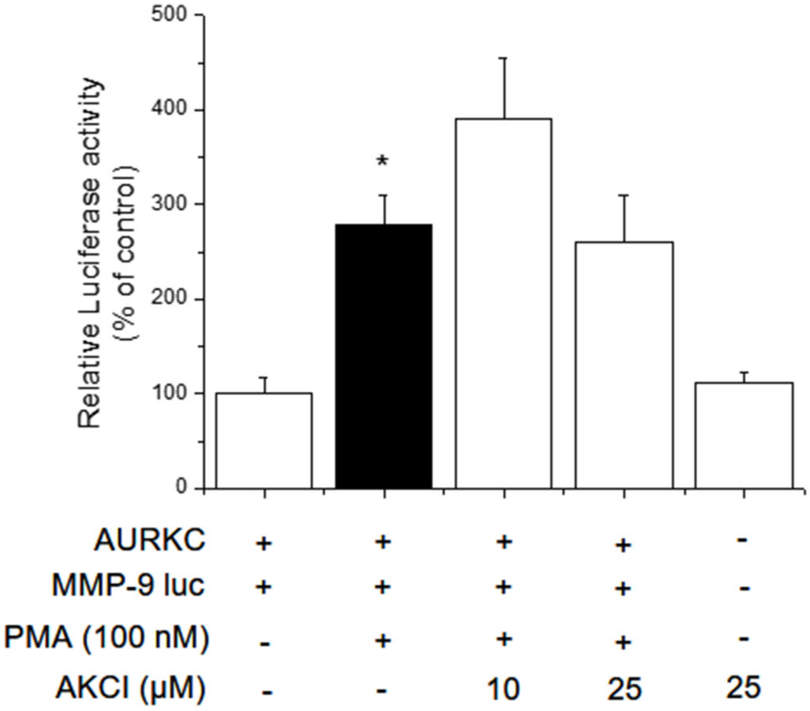

Supplementary Figure 7: Effects of AKCI on MMP-9 transcriptional activity in MDA-MB-231 cells. Cells were transfected with MMP-Luc and pCMV-β, and then cultured with AKCI for 24 h, harvested, and assayed for luciferase activity. Luciferase activity was normalized against β-galactosidase activity, and is expressed as the proportion of activity detected relative to the vehicle control. * $P < 0.01$, significantly different from control as determined by analysis of variance (Newman–Keuls test).
